# Supplementary material for: COVID-19 sentiment analysis using college subreddit data
Source: PLoS One. 2022 Nov 4;17(11):e0275862. doi: 10.1371/journal.pone.0275862 (PMC9635711; doi:10.1371/journal.pone.0275862)
Supplement: S1 File — (PDF) [file pone.0275862.s001.pdf]

Supporting Information for "COVID-19 sentiment analysis using college subreddit data"

Tian Yan[1], Fang Liu[1*]

1 Applied and Computational Mathematics and Statistics, University of Notre Dame, Notre Dame, IN 46556, United States

* fliu2@nd.edu

S1 File. The file contains the data and code information and the supporting table

Data and Code The data we collected from Reddit for this study include 1) userID, 2) time of a posted message, 3) content of a message, 4) dyadic relation between two messages in terms of whether one is a reply to the other. There is no information in the collected data that allows direct identification of a user in the data. The information in 'userID' was not used in our model training or methodological development and was replaced by dummy integers after the data were downloaded.

The data collection is in compliance with the Reddit privacy policy [1], Reddit user agreement [2], and Reddit API's term of use [3]. The data are anonymized in the sense that we replaced the actual Reddit userIDs in the data with dummy IDs (integers 1, 2, ...), further limiting potential privacy risk (e.g., through record linkage) without losing information for learning task based on the data. The unprocessed data with actual Reddit userIDs replaced by dummy IDs are available at `https://drive.google.com/file/d/13PzwBAjyI4VYpCEVye6fU3gGsDvdnrBY/view?usp=sharing` and the RoBERTa-processed data and scores are available at `https://drive.google.com/file/d/1RRi5o1JCWeuS-dfhNOuxsk7JxJXKBOuL/view?usp=sharing`.

The code for this study can be download from `https://github.com/AlvaYan/Sentiment-Analysis-GNN-During-COVID19`). The Python code for the RoBERTa framework that we applied is adapted from [**?**] and is available at `https://huggingface.co/cardiffnlp/twitter-roberta-base-sentiment`; the Python code for training the GAT NN is adapted from [**?**] (`https://github.com/Jhy1993/HAN`).

Table Estimated effects of pandemic and in-person learning on odds ratios of negative sentiment via GLMM based on the predicted outcome labels by RoBERTa and GAT.

labelling method	Effect of pandemic			Effect of in-person learning in 2020		
	Factor	odds ratio	p-Value	Factor	odds ratio	p-Value
GAT	**Year 2020**	**1.310**	$\mathbf{< 0.001}$	**In-Person**	**1.562**	**0.011**
	Small City	1.106	0.410	Small City	1.231	0.229
	Private	0.723	0.051	Private	0.686	0.052
RoBERTa	**Year 2020**	**1.196**	$\mathbf{< 0.001}$	**In-Person**	**1.414**	**0.049**
	Small City	1.073	0.641	Small City	1.148	0.350
	Private	0.839	0.219	Private	0.818	0.249

References

1. Reddit Privacy Policy. https://www.redditinc.com/policies/privacy-policy.
2. Reddit User Agreement. https://www.redditinc.com/policies/user-agreement-september-12-2021.
3. Reddit API Terms of Use. https://docs.google.com/forms/d/e/1FAIpQLSezNdDNK1-P8mspSbmtC2r86Ee9ZRbC66u929cG2GX0T9UMyw/viewform.
